# Dissecting the association of autophagy-related genes with cardiovascular diseases and intermediate vascular traits: A population-based approach

**DOI:** 10.1371/journal.pone.0214137

**Published:** 2019-03-25

**Authors:** Eliana Portilla-Fernandez, Mohsen Ghanbari, Joyce B. J. van Meurs, A. H. Jan Danser, Oscar H. Franco, Taulant Muka, Anton Roks, Abbas Dehghan

**Affiliations:** 1 Department of Epidemiology, Erasmus University Medical Center, Rotterdam, The Netherlands; 2 Department of Internal Medicine, Division of Vascular Medicine and Pharmacology, Erasmus University Medical Center, Rotterdam, The Netherlands; 3 Institute of Social and Preventive Medicine, University of Bern, Bern, Switzerland; 4 Department of Epidemiology and Biostatistics, Imperial College London, London, England; Medical University Innsbruck, AUSTRIA

## Abstract

Autophagy is involved in cellular homeostasis and maintenance and may play a role in cardiometabolic health. We aimed to elucidate the role of autophagy in cardiometabolic traits by investigating genetic variants and DNA methylation in autophagy-related genes in relation to cardiovascular diseases and related traits. To address this research question, we implemented a multidirectional approach using several molecular epidemiology tools, including genetic association analysis with genome wide association studies data and exome sequencing data and differential DNA methylation analysis. We investigated the 21 autophagy-related genes in relation to coronary artery disease and a number of cardiometabolic traits (blood lipids, blood pressure, glycemic traits, type 2 diabetes). We used data from the largest genome wide association studies as well as DNA methylation and exome sequencing data from the Rotterdam Study. Single-nucleotide polymorphism rs110389913 in *AMBRA1* (p-value = 4.9×10^−18^) was associated with blood proinsulin levels, whereas rs6587988 in *ATG4C* and rs10439163 in *ATG4D* with lipid traits (*ATG4C*: p-value = 2.5×10^−15^ for total cholesterol and p-value = 3.1×10^−18^ for triglycerides, *ATG4D*: p-value = 9.9×10^−12^ for LDL and p-value = 1.3×10^−10^ for total cholesterol). Moreover, rs7635838 in *ATG7* was associated with HDL (p-value = 1.9×10^−9^). Rs2447607 located in *ATG7* showed association with systolic blood pressure and pulse pressure. Rs2424994 in *MAP1LC3A* was associated with coronary artery disease (p-value = 5.8×10^−6^). Furthermore, we identified association of an exonic variant located in *ATG3* with diastolic blood pressure (p-value = 6.75×10^−6^). Using DNA methylation data, two CpGs located in *ULK1* (p-values = 4.5×10^−7^ and 1×10^−6^) and two located in *ATG4B* (2×10^−13^ and 1.48×10^−7^) were significantly associated with both systolic and diastolic blood pressure. In addition one CpG in *ATG4D* was associated with HDL (p-value = 3.21×10^−5^). Our findings provide support for the role of autophagy in glucose and lipid metabolism, as well as blood pressure regulation.

## Introduction

Autophagy is a highly conserved catabolic process involved in the degradation of long-lived proteins and dysfunctional organelles [1]. Under conditions of oxidative stress, hypoxia and nutrient deprivation, autophagy is activated as a key mechanism of cell survival by degradation and recycling of damaged organelles and protein aggregates [2]. A disturbance in removal of these non-functional cellular components is a general impairment in housekeeping of the cell, and could lead to important phenotypic changes at cell and tissue levels [3]. Several studies, mainly based on animal models, have provided evidence about the role of autophagy in the progression of ageing and in particular in atherosclerosis, inflammation, and cardiovascular disease [2, 4–8]. A number of autophagy-related mechanisms have been studied for cell survival and in some instances the role of genetic variants involved in molecular mechanisms of autophagy were investigated in relation to cardiometabolic health in animal models [9]. Systemic knockout of autophagy-related genes (*ATG)* in mice has shown the role of dysfunctional autophagy in hyperglycemia [10], hypoinsulinemia and increased basal Ca2+ concentrations in vascular smooth muscle cells [11, 12]. However, this association has not been studied at population level. Genetic and epigenetic variations in *ATG* could affect the autophagy process in human cells, modify certain metabolic traits and eventually cause susceptibility to cardiometabolic disorders [13, 14]. In this study, by using Genome-Wide Association Studies (GWAS) data and data from the Rotterdam Study, a population-based prospective cohort study, we aimed to examine the association of genetic and epigenetic variation in *ATG* with intermediate vascular traits and cardiovascular outcomes.

## Materials and methods

The Rotterdam Study has been approved by the Medical Ethics Committee of the Erasmus MC (registration number MEC 02.1015) and by the Dutch Ministry of Health, Welfare and Sport (Population Screening Act WBO, license number 1071272-159521-PG). The Rotterdam Study has been entered into the Netherlands National Trial Register (NTR; www.trialregister.nl) and into the WHO International Clinical Trials Registry Platform (ICTRP; www.who.int/ictrp/network/primary/en/) under shared catalogue number NTR6831. All participants provided written informed consent to participate in the study and to have their information obtained from treating physicians.

### Identification of autophagy-related genes

**Fig 1:**
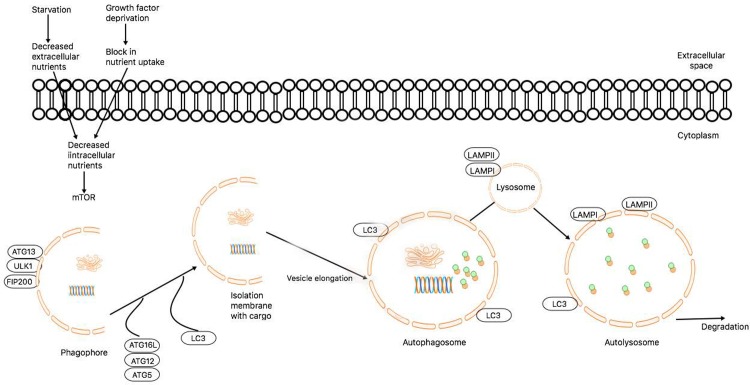
Molecular pathway of autophagy. Autophagy activation is controlled by several factors, including amino acid and nutrient deprivation, which stimulates mTOR-related pathways. Upon mTOR inhibition by starvation or rapamycin, *ATG13*/ULK1/FIP200 form a stable complex, essential for autophagy activity. Highly coordinated autophagy-related proteins are recruited to the phagosphore to participate in the autophagosome formation. *ATG7* is one of the crucial regulators of this process. Lipidation of LC3-I known as LC3-II is attached to the autophagosome membrane and is widely used to monitor autophagy induction. When autophagosome formation is completed, the fusion between autophagosome and lysosome is mediated by lysosomal membrane protein LAMP-2. *ATG12* conjugation to *ATG5* is a crucial step in autophagosome formation. *ATG7* regulates ubiquitin-like reaction that mediates this mechanism and subsequent processes in the autophagy machinery.

To select the set of autophagy-related genes to be studied, a literature search was performed and was updated by August 2018 in PubMed using the MeSH terms: Autophagy, genome-wide association studies, candidate gene studies, and knockout experimental models. We identified a total of 30 genes related to autophagy and associated pathways. We further included the genes in the pathway analysis done by QIAGEN’s Ingenuity Pathway Analysis Software (IPA, http://www.ingenuity.com, Fig 1) to determine genes enriched for autophagy. Core Analysis implemented by IPA was used to interpret the role of these genes in the context of biological processes, pathways and molecular networks. IPA uses right-tailed Fisher exact test to identify enriched canonical pathways and diseases associated to these genes. A p-value < 0.05 was considered significant. A set of 21 genes showed significant enrichment for autophagy (p-value = 1.33×10^−40^) (Table 1). We studied genetic variants within the genes and those located within 10 MB upstream and downstream of the gene. We excluded SNPs with a reported minor allele frequency (MAF) less than 1% in NCBI.

**Table 1:** Autophagy-related genes and the number of SNPs selected for this study.

Gene	Pathway	Chromosome	Independent SNPs
*AMBRA1*	Autophagic vacuole formation	11	34
*ATG1*	Molecular activation of autophagy	12	18
*ATG3*	Protein transport. Protein ubiquitination	3	27
*ATG4B*	Autophagic vacuole formation	2	8
*ATG4C*	Autophagic vacuole formation	1	43
*ATG4D*	Autophagic vacuole formation	19	19
*ATG5*	Autophagic vacuole formation	6	49
*ATG7*	Protein transport. Protein ubiquitination	3	98
*ATG9A*	Autophagic vacuole formation	2	11
*ATG9B*	Autophagic vacuole formation	7	16
*ATG10*	Protein transport	5	101
*ATG12*	Autophagic vacuole formation	5	12
*ATG16L1*	Autophagic vacuole formation	2	43
*ATG16L2*	Protein transport	11	8
*BECLIN-1*	Regulation of autophagy	17	2
*DRAM1*	Autophago-lysosome formation	12	43
*GABARAP*	Autophagic vacuole formation	17	7
*GABARAPL1*	Autophagic vacuole formation	12	23
*GABARAPL2*	Autophagic vacuole formation	16	11
*MAP1LC3A*	Autophagic vacuole formation	20	16
*MAP1LC3B*	Autophagic vacuole formation	16	15

### Genetic association

For each gene, we studied the association of the genetic variants intermediate vascular traits and cardiovascular outcomes. We extracted the summary statistics for the association of the selected SNPs with traits and diseases from the most recent GWAS meta-analysis of 12 traits: fasting glucose, fasting insulin, fasting proinsulin [15–18], type 2 diabetes [19], systolic blood pressure (SBP), diastolic blood pressure (DBP) [20], total cholesterol (TC), triglycerides (TG), HDL cholesterol (high density lipoprotein), LDL cholesterol (low density lipoprotein) [21] and coronary artery disease (CAD) [22]. Details of the consortia included are provided in S1 Note and number of subjects included in each study is shown in Table 2.

**Table 2:** Description of GWAS meta-analysis on cardiometabolic disorders.

Trait	Consortium	Sample size
Coronary artery disease	UK Biobank/CardiogramplusC4D [23]	122,733 cases / 424,528 controls
Fasting glucose	MAGIC [15]	133,010
Fasting insulin	MAGIC [16]	108,557
Proinsulin	MAGIC [17]	10,701
Type 2 diabetes	DIAGRAM [19]	26,676 cases / 132,532 controls
Blood pressure	UK biobank [20]	140,000
Total cholesterol	ENGAGE [21]	100,184
Triglycerides	ENGAGE [21]	96,598
HDL cholesterol	ENGAGE [21]	99,900
LDL cholesterol	ENGAGE [21]	95,454

MAGIC: Meta-analysis of Glucose and Insulin-related traits Consortium; DIAGRAM: Diabetes Genetics Replication and Meta-analysis; CARDIOGRAMplusC4D: Coronary Artery Disease Genome wide replication and Meta-analysis plus Coronary Artery Disease; HDL: High density lipoprotein; LDL: Low density lipoprotein

### Exonic variants

We studied the association of exonic variants and DNA methylation status in *ATG* with lipid, glycemic and intermediate vascular traits using data from the Rotterdam Study. The Rotterdam Study is an ongoing prospective, population-based cohort study started in 1990 including subjects from Ommoord district in the city of Rotterdam (The Netherlands). The objectives and details of the study are described elsewhere [24]. The Rotterdam Study comprises a total of 14,926 subjects aged 45 years and over who were recruited in three sub cohorts in 1989–1993, 2000–2001, and 2006 [24]. Genomic DNA was extracted from peripheral blood mononuclear cells. Whole-Exome sequencing (WES) was performed at the Rotterdam Genomics Core in Erasmus Medical Centre. WES was conducted on 2,998 paired-end sequenced samples using the Illumina hiSeq2000 (2×100bp reads). Indels and single nucleotide variants were filtered out and evaluated using GAKs Variant Evaluation; variants with a call rate <0.97, duplicate samples, duplicate variants and >5% of missing genotypes were considered as exclusion criteria. A total of 2628 samples passed through all technical quality control and GATKs Haplotype Caller was used to call SNPs and indels simultaneously. Annovar software tool was used to functionally annotate each genetic variant. Each variant was coded as 0, 1 and 2 representing two reference alleles, one reference allele and one mutated allele and two mutated alleles, respectively. Based on the functional annotation, we identified a total of 13 loss of function (LoF) variants defined as changes in DNA sequence predicted to completely disrupt the formation and/or function of a protein.

### DNA methylation data collection and normalization

DNA was extracted from whole peripheral blood (stored in EDTA tubes) by standardized salting out methods. The Infinium HumanMethylation450 BeadChip array was employed to determine the gene methylation status using DNA from whole blood samples of 468 individuals from third visit of the second sub-cohort, 731 individuals from first visit of the third sub-cohort, and 251 individuals from second visit of the third sub-cohort (no-overlap). The array covers approximately 485,577 methylation sites (in 99% of Ref Seq genes approximately) with an average of 17.2 CpG sites per gene region. In short, samples (500 ng of DNA per sample) were first bisulfite treated using the Zymo EZ-96 DNA-methylation kit (Zymo Research, Irvine, CA, USA). Next, they were hybridized to the arrays according to the manufacturer’s protocol. The methylation percentage of a CpG site was reported as a beta-value ranging between 0 (no methylation) and 1 (full methylation). The quality control of DNA methylation data was conducted for both sample and probe based on a p-value threshold for gene detection of 0.01. Data normalization was done based on color bias correction of methylated and unmethylated signals, per plate and separate color background adjustment. Outliers beyond the 3^rd^ quartile were excluded and the final dataset comprised 463,456 CpG sites.

### Clinical traits and outcomes

Lipid measurements were carried out using venous blood samples obtained from all participants of the Rotterdam Study. Total cholesterol, high-density lipoprotein cholesterol, and triglycerides were measured on the COBAS 8000 Modular Analyzer (Roche Diagnostics GmbH). Low density lipoprotein cholesterol levels were estimated indirectly from measurements of TC, HDL, and TG by means of the Friedewald equation [25]. The corresponding interassay coefficients of variation was <2.1%.

During each visit, blood pressure was measured twice in the right arm using a random-zero sphygmomanometer, in sitting position, after a resting period of 5 minutes. After 2006 Omron M6 Comfort and Omron M7 devices were used. The measurements were taken in duplicate. The average of the 2 measurements was used in the analyses. A qualified physician at the research center collected data on indication for use of BP-lowering medication during the interview.

Glucose and insulin were measured from venous blood samples. Fasting insulin and glucose were measured on the COBAS 8000 Modular Analyzer (Roche Diagnostics GmbH). The interassay coefficients of variations are <8% and <1.4% for insulin and glucose respectively. Type 2 diabetes was defined according to recent WHO guidelines, as a fasting blood glucose ≥ 7.0 mmol/L, a non-fasting blood glucose ≥ 11.1 mmol/L (when fasting samples were absent), or the use of blood glucose lowering medication [26]. Information regarding the use of diabetes medication was collected from both structured home interviews and linkage to pharmacy records [27].

### Statistical analysis

#### SNP pruning and Genome-wide association studies look up

We found 5398 SNPs in the 21 identified autophagy genes. Linkage disequilibrium (LD)-based SNP pruning implemented in PLINK software was applied using a genetic correlation threshold of 0.5 to calculate the number of independent SNP per gene [28]. Using LD pruning, we identified 604 independent SNPs in 21 genes directly involved in autophagy pathway. We applied Bonferroni correction for 604 SNPs and 4 traits. The study-wide significance threshold was set at 2.07×10^−5^ (0.05/604 SNPs×4 traits).

#### Exome sequencing

We used Seqmeta, an R package to perform region-based tests of rare DNA variants, to conduct multiple regression models in order to determine association between LoF variants and intermediate vascular traits including age and sex as a covariates. The package allows to obtain the scores and MAF for each gene with the function prepScores and to calculate both gene-based and single variant-based associations based on the prepScores output [29, 30]. The significance level was based on Bonferroni correction to adjust for multiple comparisons according to the number of variants and traits being tested. Here, we set a p-value threshold as 9.62×10^−4^ (0.05/(4 traits×13 LoF))

#### Epigenome-wide association studies

We conducted epigenome wide association studies (EWAs) on blood pressure, glucose, insulin, HDL, LDL, triglycerides and total cholesterol. Beta-values to quantify the DNA methylation levels were used in a linear mixed-effect regression model with the outcomes of interest as a dependent variable. We fit the primary regression model adjusting by age and sex, cell counts, technical covariates and smoking. Further adjustments including, hypertensive or lipid lowering treatment, prevalent diabetes mellitus were employed for blood pressure and lipids levels respectively. We combined the results of the three sub-cohorts by conducting a fixed effect meta-analysis using the inverse variance method implemented in METAL [31]. We established the target set of *ATG-*CpGs considering the CpG islands found in upstream, downstream and promoter regions of each gene. CpG sites per gene were obtained using the UCSC genome browser (http://genome.ucsc.edu/). Statistically significant association was set at 5.63×10^−5^ based on Bonferroni correction for 296 CpG sites and three trait groups (glycemic traits, lipids and blood pressure). Next to the identification of epigenetic marks in *ATG* genes, we extended our search of methylation quantitative trait loci (meQTLs) by the identification of both *cis* and *trans* associated SNPs using a large meQTLs dataset from mQTLdb resource (http://www.mqtldb.org/) [32].

## Results

### Genetic association

The results of genetic association of the SNPs and cardiometabolic traits are presented in S1 Table. The lead significant variants are presented at Table 3. Six SNPs at three genes, *AMBRA1* (Lead SNP: rs11038913), *ATG13* (rs8914), and *ATG16L1* (rs4944804) passed the significant threshold for association with pro-insulin serum levels. Total cholesterol level was significantly associated with 34 SNPs at *ATG4D* (rs10439163) and one SNP at *ATG4C* (rs6587988). HDL-cholesterol was associated with 41 variants at *ATG7* (rs7635838). One SNP at *ATG4D* (rs10439163) was associated with LDL-cholesterol and the one at *ATG4C* (rs6587988) was further associated with triglyceride levels. Moreover, 56 SNPs and 38 SNPs located in *ATG7* were associated with SBP and pulse pressure, respectively. Two SNPs (rs2424994 and rs6088521) located at *MAP1LC3A* showed an association with CAD.

**Table 3:** SNPs in autophagy genes with the most significant association with cardiometabolic traits and diseases.

SNP	Gene	Trait/Disease	P-value	MAF
rs7635838	*ATG7*	HDL-cholesterol	1.9×10^−9^	0.49
rs2447607		Systolic blood pressure	3.2×10^−8^	0.40
		Pulse pressure	3.5×10^−7^	0.40
rs10439163	*ATG4D*	LDL-cholesterol	9.9×10^−12^	0.43
		Total cholesterol	1.4×10^−10^	0.43
rs7255312		Coronary artery disease	6.1×10^−6^	0.10
rs6587988	*ATG4C*	Triglycerides	3.1×10^−18^	0.24
		Total cholesterol	2.5×10^−15^	0.24
rs11038913	*AMBRA1*	Fasting proinsulin	4.9×10^−18^	0.08
rs8914	*ATG13*		7.1×10^−18^	0.03
rs4944804	*ATG16L1*		8.9×10^−18^	0.13
rs6088521	*MAP1LC3A*	Coronary artery disease	4.1×10^−6^	0.50

MAF: minor allele frequency; HDL: High-density lipoprotein; LDL: low-density lipoprotein. Significance threshold: 2.07×10^−5^

In addition to this significant association, we also observed borderline significant association between SNPs at *ATG7* with SBP, *ATG4B* with fasting proinsulin, and *MAP1LC3A* with HDL-cholesterol. No associations were found between *ATG* and type 2 diabetes.

### Loss of function variants

We used data from 2,628 participants of the first sub-cohort of the Rotterdam Study. Baseline characteristics of the individuals are presented in S2 Table. We identified 13 LoF stop-gain or splicing mutations in *ATG4C*, *ULK1*, *GABARAPL2*, *GABARAP*, *ATG4D*, *ATG3*, *ATG10* and *ATG5* (Table 4). We used single variant-based analysis implemented in SeqMeta to assess the association of LoF mutations with intermediate vascular traits. We identified one exonic variant in *ATG3* associated with DBP and fasting glucose (p-value for SBP: 0.054). The C/A variant found in this gene is considered a nonsense mutation and is related with stop-gain function of the protein. We also found a nominally significant association between an exonic variant in *ULK1* and triglycerides (p-value = 0.0012). The results of association between these variants and studied traits and diseases are mentioned in S3 Table.

**Table 4:** LOF variants associated with intermediate vascular traits.

Variant	Gene	Trait	P-value
3:112277264	*ATG3*	Diastolic blood pressure	6.8×10^−6^
		Fasting glucose	0.043
12:132404136	*ULK1*	Triglyceride	1.3 ×10^−3^
12:132404136	*ULK1*	Total cholesterol	0.18
12:132404136	*ULK1*	HDL-cholesterol	0.08
12:132404136	*ULK1*	Fasting insulin	0.65
12:132404136	*ULK1*	LDL-cholesterol	0.19
12:132404136	*ULK1*	Systolic blood pressure	0.05

Significance threshold: 9.61×10^−4^

### DNA methylation at autophagy genes

In total, 1450 individuals with a mean±SD age of 60.6 ± 5.3 years were included in the analysis. The association of the studied CpGs and all traits and diseases are presented in S4 Table. In a fully adjusted model, we found differential methylation at *ATG4B* and *ULK1* were associated with blood pressure (Table 5). Hypermethylation in these CpGs were associated with lower SBP/DBP. On the other hand, no significant associations between DNA methylation and glycemic and lipid traits were found in the study population. Using mQTLdb database, we identified five *trans-*meQTLs associated with cg02710553. The variants were located in an intergenic region close to *MAP3K7*. Furthermore, 23 *trans-*meQTLs associated cg06006530 were identified close to *CDH18* gene. meQTLs of cg02710553 were found to be associated with CAD at nominal significance level (p-value = 0.02).

**Table 5:** DNA methylation, blood pressure and HDL.

CpG site	Gene	Trait	Effect	P-value*
cg08462942	*ATG4B*	Systolic blood pressure	-0.00026	2.0×10^−13^
cg06006530			-0.0019	1.5×10^−7^
cg02710553	*ULK1*	Systolic blood pressure	-0.0018	4.5×10^−7^
		Diastolic blood pressure	-0.0018	1.0×10^−6^
cg10819350	*ATG4D*	HDL-cholesterol	-0.0158	3.2×10^−5^

*Adjusted for age, sex, cell counts, batch effects, anti-hypertensive and lipid-lowering medication

HDL: high density lipoprotein, significance threshold: 5.63×10^−5^

## Discussion

In this study we used genetic and epi-genetic factors to assess the role of autophagy genes in cardiometabolic traits and disorders at population level. To this end, we utilized a number of genetic variants, LoF variants and DNA methylation in autophagy related genes. All approaches indicated associations between autophagy genes and certain cardiometabolic traits, mainly blood pressure, lipid levels and proinsulin levels but not coronary artery disease.

Our findings on the association between genetic variants in *AMBRA1*, *ATG13* and *ATG16L1* and proinsulin levels are in agreement with previous evidence reporting autophagy deficiency as an important determinant in the pathogenesis of insulin resistance and diabetes [33]. AMBRA1 participates in the activation of beclin-1-regulated autophagy and favors the autophagosome core complex with the participation of other ATG proteins such as ATG13 and ATG16L1 [34]. The upstream autophagy-signaling network controlled by AMBRA1 is crucial for the metabolic response to a vast number of stress stimuli, ranging from starvation to hypoxia or DNA damage. Dysfunctional autophagy has been suggested as a key process related to an impaired proinsulin/insulin homeostasis observed in pancreatic beta cells from experimental models [35]. Conditional knockout of *ATG7* in high-fat-fed C57BL/6 mice has resulted in declined insulin secretion, impaired glucose tolerance and degeneration of pancreatic islets [36]. In autophagy-deficient cells, the insulin secretion is restrained enabling the proinsulin accumulation in secretory granules and increased secretion in response to stimuli [37]. At population level, in line with our findings, an exome array analysis conducted in 8229 individuals showed association between genetic variants in *AMBRA1* and *ATG13* with fasting proinsulin concentrations [38]. The role of impaired autophagy in proinsulin degradation has highlighted the importance of the autophagy pathway on the design of novel therapeutic strategies, aimed to manipulate proinsulin clearance as means to increase the insulin secretion in diabetic population.

Our findings on common genetic variants in *ATG* and blood lipids support the hypothesis that autophagy may play a role in dyslipidemia. The role of impaired autophagy and lipid metabolism has been established from the identification of significant increased levels of hepatic triglycerides and cholesterol content in hepatocyte-specific knockout mice of *ATG5* [39]. Moreover *ATG7* knockout mice models have displayed severe morphological abnormalities in the structure of white adipocytes, as well as an aggravated insulin resistance with increased lipid content and inflammatory changes [40, 41]. In summary, upregulation of autophagy leads to a decrease of triglycerides and cholesterol in plasma, reduced lipid store as well as LDL oxidation and free fatty acid B-oxidation and an increase of folding and traffic proteins [42]. Further investigations are needed to clarify this association and potential pathways linking autophagy with blood lipids levels. Recent findings of a critical role for macroautophagy in the metabolism and storage of cellular lipids have now suggested that alterations in autophagy may mediate human disorders marked by excessive cellular lipid stores.

In contrast, mice with endothelial specific deletion of *ATG7* have shown normal blood pressure and normal vessel architecture compared to wild types [43]. We further identified a suggestive association between LoF variants annotated in *ATG3* and glucose levels. It has been shown that glycogen autophagy in newborns serves as a mechanism of glucose homeostasis [44]. In adult animals, the administration of glycogen autophagy-inhibiting insulin triggers a reduced rate of breakdown of liver glycogen by autophagy [45]. Impaired mechanisms related to autophagosomal glucose production and the influence of gluconeogenesis may lead to a dissociation of gluconeogenic glucose production from blood glucose levels [46]. On the other hand, we found no association between changes in DNA methylation patterns and glycemic traits. This might be explained by the fact that methylation is a tissue-specific process. Therefore, SNPs could be operating independently from methylation patterns.

We studied DNA methylation at autophagy-related genes and found differential patterns at *ULK1* and *ATG4B* associated with blood pressure levels. Increased blood pressure is determined by a complex machinery regulated, among others, by the renin-angiotensin system (RAS) [47]. The interaction between autophagy and RAS has been previously examined. Porrello et al, provided the first evidence for an interplay between these two mechanisms in cardiomyocytes. This study reported that rat cardiomyocytes developed and augmented (via the angiotensin II receptor type 1) or inhibited (via the angiotensin II receptor type 2) autophagic response on stimulation by angiotensin II [48]. The association between autophagy and blood pressure found in our study could be explained by its interaction with RAS and its role in hypertension. Given the fact that autophagy and RAS are both involved in many pathophysiological processes, further investigation is warranted to better understand the molecular mechanism behind this interaction and its role in pathological conditions. In addition, we found an association between DNA methylation in *ATG4D* and HDL levels. *ATG4D* is known to participate in the delipidation of GABARAP-L1, whereas the silencing of *ATG4D* abrogates GABARAP-L1 autophagosome formation [49]. Experimental evidence of the role of this gene on lipid transport/metabolism is currently lacking.

Epi-genetic associations are subject to confounding and reverse causation. One approach to overcome these biases in molecular epidemiology is to use genetic instruments. In this study, we found that meQTL at cg02710553 was associated with CAD. This might indicate that the association of cg2710553 and CAD is causal.

We used both genomics and epi-genomics data to examine the role of autophagy related genes in cardiometabolic traits and diseases. Although we have several significant association, we did not find a consistent association with a certain gene or a certain trait across all approaches. It should be noted that genomic and epi-genomic approaches are not always pointing to the same gene. Moreover, it is not a surprise if a different gene in a certain pathway has more or less importance for a certain trait. The high complexity of the autophagy mechanism may be a major contributor of the heterogeneity of the findings in our results [50].

Our study has several strengths. First, we used data from largest GWAS, which has provided the best statistical power that could be achieved. Second, we used both genetic and epi-genetic approaches towards the research question. Third, we used exome sequence data, which in contrast to GWAS, is not based on a studying a proxy variant. Our study also had several limitations. First, the sample size for exome sequence analysis was much smaller than the GWAS, thus the statistical power was significantly lower. Loss of function variants are mostly found in low frequency, indicating that they are enriched for mildly deleterious polymorphisms suppressed by negative natural selection [51]. Therefore, smaller populations might also provide enough statistical power to detect their effect, however, this could not be ruled out that lack of association in our analysis might be a results of small sample size.

Second, DNA methylation pattern is tissue/cell line specific. As it is common in epidemiologic studies we used DNA methylation in whole blood, a cell type mixture, which might not be relevant for some of the traits. In our study DNA methylation was captured from the assessment of leukocytes. Although leukocytes are not relevant tissue for some of the traits that we have assessed in our analysis, they are the main tissue available in large scale in epidemiologic studies and evidence has demonstrated that methylation patterns might correlate between blood and the relevant tissues, suggesting that the use of blood tissue could yet be informative [52–55]. However, such findings should be validated in subsequent studies.

In conclusion, this study is the first to examine the role of autophagy-related genes in intermediate vascular traits using a population-based approach. We have characterized the role of common and rare genetic variants as well as epigenetic variations of autophagy-related genes on several traits. Despite the heterogeneity of our findings across approaches employed and traits evaluated, we found many associations between autophagy genes and cardiometabolic traits and diseases. The integral approach covered by this study could contribute to further analysis evaluating the role of autophagy in other human diseases and traits, as well as the design of experimental studies targeting other autophagy-related genes and/or associated pathways.

## Supporting information

S1 Note Brief description of the consortia included in this study. (DOCX)

S1 Table Significance levels obtained for *ATG* variants in relation with each trait under study. These p-values were obtained from the summary statistics provided by the consortia.
(XLS)

S2 Table Baseline characteristics of the participants from Rotterdam Study included in this paper. (DOCX)

S3 Table P-values reported for the association between the traits evaluated and LOF variants. (XLS)

S4 Table P-values reported for the association between the traits evaluated and CpG sites in *ATG*. (XLS)
