# Supplementary material for: Dissecting the association of autophagy-related genes with cardiovascular diseases and intermediate vascular traits: A population-based approach
Source: PLoS One. 2019 Mar 25;14(3):e0214137. doi: 10.1371/journal.pone.0214137 (PMC6433264; doi:10.1371/journal.pone.0214137)
Supplement: S1 Note — (DOCX) [file pone.0214137.s001.docx]

**Supplementary note**

- **MAGIC**. The Meta-analyses of glucose and insulin-related traits consortium (MAGIC) aims to unify data from GWAS to identify loci associated with metabolic traits including fasting glucose, fasting insulin, fasting proinsulin, HbA1c, HOMA-B, and HOMA-IR from up to 133000 individuals. Through this effort, dozens of loci influencing these traits have been identified.
- **DIAGRAM**. The diabetes genetics replication and meta-analysis (DIAGRAM) consortium represents a collaborative effort to characterize the genetic basis of type 2 diabetes combining data from large-scale genetic studies. The latest GWAS meta-analysis has included 26676 subjects with type 2 diabetes and 132532 controls.
- **ENGAGE***.* ENGAGE (European Network for Genetic and Genomic Epidemiology) is a research consortium developed under the 7th Framework Programme-Health Theme. The ENGAGE Consortium has brought together 24 leading research organizations aiming to translate data from large-scale research in genetic and genomic epidemiology from European (and other) population cohorts into information relevant to future clinical applications. The Consortium has integrated and analyzed one of the largest ever human genetics dataset (more than 80,000 genome-wide association scans and DNAs and serum/plasma samples from over 600,000 individuals) and has enabled researchers to identify large numbers of novel susceptibility genes that influence metabolic, behavioural and cardiovascular traits among others
- **CARDIOGRAMplusC4D and UK biobank**. CARDIOGRAMplusC4D consortium aims to identify risk loci for CAD and myocardial infarction from multiple large-scale genetic studies. UK Biobank participants Participants were recruited with an age range of 40-69 years of age that registered with a general practitioner of the UK National Health Service (NHS). Between 2006–2010, in total 503,325 individuals were included. All study participants provided informed consent and the study was approved by the North West Multi-centre Research Ethics Committee. Detailed methods used by UK Biobank have been described elsewhere. The data referred in this paper was produced by merging data from both consortium allowing the analysis of 122733 CAD cases and 424528 controls
