# Supplementary material for: Dissecting the association of autophagy-related genes with cardiovascular diseases and intermediate vascular traits: A population-based approach
Source: PLoS One. 2019 Mar 25;14(3):e0214137. doi: 10.1371/journal.pone.0214137 (PMC6433264; doi:10.1371/journal.pone.0214137)
Supplement: S2 Table — (DOCX) [file pone.0214137.s003.docx]

Supplementary table s2. Baseline characteristics of RSI and RSIII cohorts

| Characteristic | RSI | RSIII-1 | RSIII-2 | RSII-3 |  |
| --- | --- | --- | --- | --- | --- |
| N* | 2628 | 731 | 251 | 468 |  |
| Age (mean±SD) | 72.8±7.4 | 59.9±8.2 | 61.14±4.36 | 60.64±3.24 |  |
| Gender (male/female) | 42/58 | 46/54 | 97/154 | 208/260 |  |
| SBP (mean±SD) | 143.9±21.1 | 138.4±59.1 | 129.71±16.3 | 138.9±19 |  |
| DBP (mean±SD) | 75.26±11.1 | 87.19±60.5 | 80.49±10.5 | 79.71±10.26 |  |
| Antihypertensive treatment, n (%) | - | 188 (26) | 39(15.53) | 77(16.45) |  |
| Fasting glucose,mmol/l (mean±SD) | 5.97±1.60 | 5.60±1.18 | 5.73±5.4 | 5.80±1.18 |  |
| Fasting insulin, mmol/l (mean±SD) | 4.89±6.07 | 5.51±4.39 | 811.8±7958.06 | 114.28±556.79 |  |
| Total cholesterol, mmol/l (mean±SD) | 5.77±1.00 | 5.56±1.07 | 5.56±1.04 | 5.84±0.93 |  |
| HDL, mmol/l (mean±SD) | 1.41±0.45 | 1.40±0.41 | 1.82±5.55 | 1.41±0.38 |  |
| Triglycerides, mmol/l (mean±SD) | 1.50±0.76 | 1.49±0.87 | 1.50±1.25 | 1.58±0.86 |  |
| LDL, mmol/l (mean±SD) | 3.69±0.87 | 3.48±0.97 | 3.1±0.80 | 3.7±0.91 |  |

*Number of subjects with either genetic or DNA methylation data available
